# GenomicScape: An Easy-to-Use Web Tool for Gene Expression Data Analysis. Application to Investigate the Molecular Events in the Differentiation of B Cells into Plasma Cells

**DOI:** 10.1371/journal.pcbi.1004077

**Published:** 2015-01-29

**Authors:** Alboukadel Kassambara, Thierry Rème, Michel Jourdan, Thierry Fest, Dirk Hose, Karin Tarte, Bernard Klein

**Affiliations:** 1 U1040, INSERM, Montpellier, France; 2 Department of Biological Hematology, CHU Montpellier, Montpellier, France; 3 U917, INSERM, Rennes, France; 4 Medizinische Klinik V, Universitätsklinikum Heidelberg and Nationales Centrum für Tumorerkrankungen, Heidelberg, Germany; 5 UFR Médecine, Université MONTPELLIER 1, Montpellier, France; University of Tokyo, JAPAN

## Abstract

DNA microarrays have considerably helped to improve the understanding of biological processes and diseases. Large amounts of publicly available microarray data are accumulating, but are poorly exploited due to a lack of easy-to-use bioinformatics resources. The aim of this study is to build a free and convenient data-mining web site (www.genomicscape.com). GenomicScape allows mining dataset from various microarray platforms, identifying genes differentially expressed between populations, clustering populations, visualizing expression profiles of large sets of genes, and exporting results and figures. We show how easily GenomicScape makes it possible to construct a molecular atlas of the B cell differentiation using publicly available transcriptome data of naïve B cells, centroblasts, centrocytes, memory B cells, preplasmablasts, plasmablasts, early plasma cells and bone marrow plasma cells. Genes overexpressed in each population and the pathways encoded by these genes are provided as well as how the populations cluster together. All the analyses, tables and figures can be easily done and exported using GenomicScape and this B cell to plasma cell atlas is freely available online. Beyond this B cell to plasma cell atlas, the molecular characteristics of any biological process can be easily and freely investigated by uploading the corresponding transcriptome files into GenomicScape.

## Author Summary

The use of DNA microarrays has emerged as a powerful tool for biomedical research to understand complex biological processes and diseases, generating large amounts of publicly available data. Most of these data remain unused by scientific community due to the lack of easy-to-use bioinformatics resources to analyze them. Here we present GenomicScape (www.genomicscape.com), a free online data-mining platform to identify quickly molecular changes during any biological process. As an example, we used GenomicScape to build a comprehensive and accessible molecular atlas of human B cell differentiation, which will be of great interest for immunologists to further understand normal and malignant B cell differentiation.

## Introduction

Genome-wide expression profile analysis with DNA microarrays has emerged as a powerful tool for biomedical research generating a huge amount of publicly available data. Unfortunately, the majority of these data are poorly used due to the lack of easy-to-use and open access bioinformatics tools to extract and visualize the most prominent information. Although statistical programming frameworks like R and Bioconductor projects provide free bioinformatics packages to analyze the data, they are difficult to use for untrained biologists or physicians, which limits the investigation of the large amounts of publicly available data. Based on our long-lasting experience of mining high throughput data to explore the biology of normal and malignant plasma cells [1–5], we have developed the current easy-to-use GenomicScape web tool (www.genomicscape.com), which allows to quickly analyze the molecular changes during a biological process such as cell differentiation or disease progression. As an illustration, we report here, the molecular portrait of human B cell differentiation using GenomicScape. The knowledge of the differentiation of B cells into plasma cells (PCs) has greatly improved, mostly using animal models [6], but this process is less known in humans because of the difficulty to access lymphoid organs and to mimic in vitro this in-vivo process involving multistep cell trafficking, cell interactions and activations. In this study, we took advantage of our previous works about germinal center (GC) B cells and plasma cells [3, 5, 7, 8] with the generation of publicly available transcriptome data of human naïve B cells (NBCs), centroblasts (CBs), centrocytes (CCs), memory B cells (MBCs), preplasmablasts (prePBs), plasmablasts (PBs), early plasma cells (EPCs) and bone marrow plasma cells (BMPCs). We show here how Genomicscape allows easily building an open access Atlas of the gene expression profiles of human naïve B cells to mature plasma cells, without any knowledge in bioinformatics. Additionally, all the analyses and figures of the manuscript can be prepared easily using GenomicScape. The current analysis and web atlas creation can be extended to any biological process given high throughput microarray data are uploaded into GenomicScape.

## Results

### Supervised analysis of the gene expression profiling of naïve B cells, centroblasts, centrocytes, memory B cells, preplasmablasts, plasmablasts, early plasma cells, and mature plasma cells

**Fig 1:**
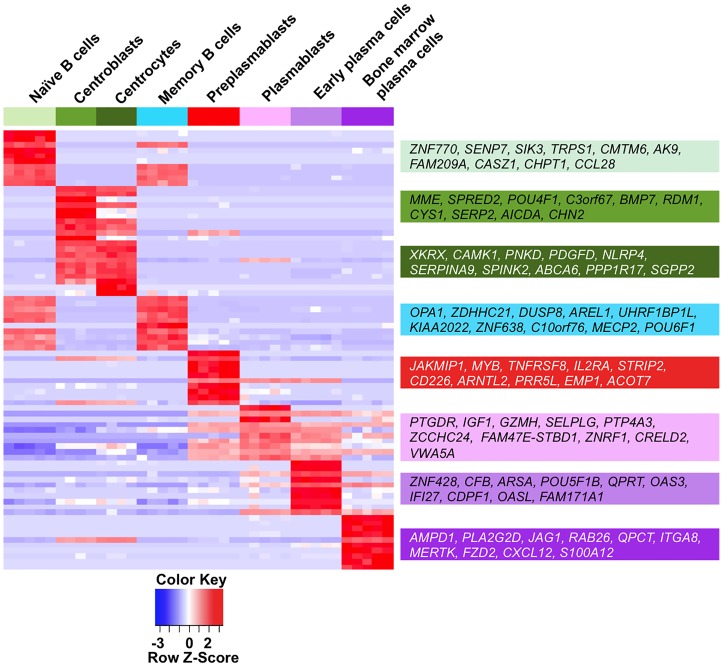
Heatmap of the top10 genes overexpressed in each of the 8 B cell to plasma cell subpopulations. The names of the top10 genes overexpressed in each cell population compared to the others are indicated on the right. Each population is highlighted by a color assigned by default by GenomicScape. The red and blue colors on the heatmap indicate over-expressed and under-expressed genes respectively and the color intensity specifies the expression level.

**Fig 2:**
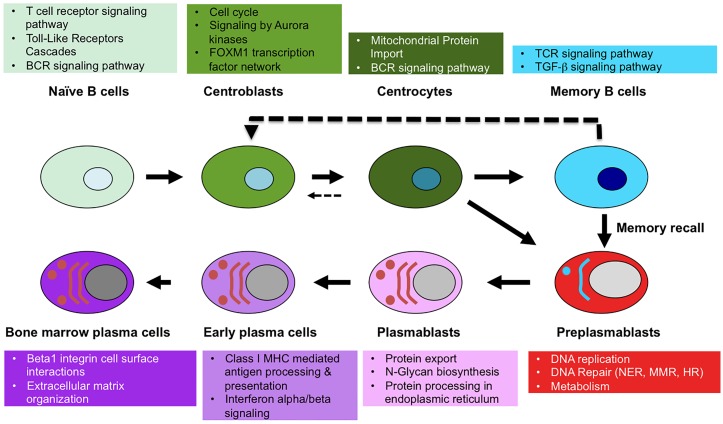
Molecular pathways enriched in the 8 B cell to plasma cell subpopulations. The molecular pathways encoded by the 9303 genes differentially expressed between the 8 cell populations were analyzed with Reactome software and the 2-3 most significant pathways for each population shown.

Publicly available gene expression data of the 8 B cell to plasma cell populations were GCRMA normalized and grouped in a “Human B cells to plasma cells GCRMA” GenomicScape file available at http://www.genomicscape.com/microarray/browsedata.php?acc=GS-DT-2. Running a SAM analysis using GenomicScape SAM tool (see S1 File) and starting from 10000 unique genes with the highest standard deviation (SD), 9303 unique genes were differentially expressed between the 8 B cell to plasma cell populations (SAM multiclass analysis, unpaired Wilcoxon statistics, fold change ≥ 2, 300 permutations, FDR = 0%). When different probe sets interrogated a same gene, the probe set with the highest standard deviation (SD) was selected. The list of 9303 genes is available at http://www.genomicscape.com/microarray/nbtopc.php and in S1 Table. Of these 9303 genes, 1740 have a SAM contrast higher in NBCs compared to the other remaining populations, 1049 in CBs, 990 in CCs, 1384 in MBCs, 1969 in PrePBs, 618 in PBs, 808 in EPCs, and 745 in BMPCs. A heatmap of the top10 genes with the highest SAM contrast in each population is shown in Fig. 1. These gene lists specific for a population can be obtained online at http://www.genomicscape.com/microarray/nbtopc.php by clicking the cell population picture in the associated scheme or the name of the population below the web table. This SAM multiclass supervised analysis identifies some genes, which are overexpressed in 2 or more populations (Fig. 1). Genes of the 9303 gene list, which are differentially expressed between 2 populations, can be identified with the “SAM” tool (http://genomicscape.com/microarray/webSam.php). The method is explained in S1 File and S1 Fig. displays the top 20 genes differentially expressed between NBCs and MBCs, CBs and CCs, prePBs and PBs, PBs and EPCs and EPCs and BMPCs. The detailed gene lists can be computed easily following S1 File. The molecular pathways encoded by the 9303 differentially expressed genes were analyzed with Reactome functional interaction cytoscape plugin (S2 Table) and the top pathways are shown in Fig. 2. Pathways enriched in NBCs are T cell receptor signaling pathway, toll-like receptors cascades and BCR signalling pathway; in CBs cell cycle, signaling by Aurora kinases and FOXM1 transcription factor network; in CCs mitochondrial protein import and BCR signaling pathway; in MBCs TCR signaling and TGF-β; in PrePBs DNA replication, DNA repair and metabolism; in PBs protein processing and export, and N-Glycan biosynthesis; in EPCs class I MHC mediated antigen processing and presentation, and interferon alpha/beta signaling; and in BMPCs beta1 integrin cell surface interactions and extracellular matrix organization proteins (Fig. 2 and S2 Table).

### Principal component analysis of genes differentially expressed between naïve B cells, centroblasts, centrocytes, memory B cells, preplasmablasts, plasmablasts, early plasma cells, and mature plasma cells

**Fig 3:**
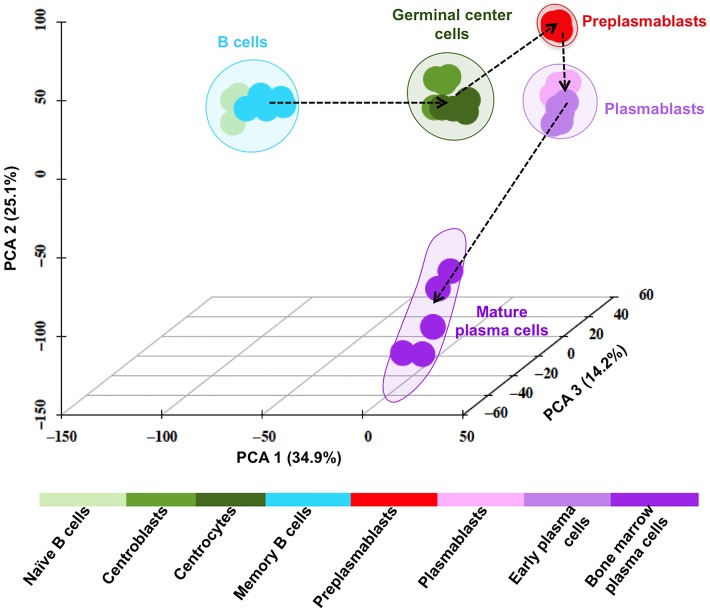
Principal component analysis of the gene expression profiles of the 8 B cell to plasma cell subpopulations. Using SAM multiclass analysis, 9303 unique genes were significantly differentially expressed between the 8 cell populations (fold change ≥ 2 and FDR = 0%). These genes classified the 8 populations into 8 distinct groups using principal component analysis and these 8 populations could be grouped into 5 major clusters: a B-cell cluster including NBCs and MBCs, a GC cluster CBs and CCs, a PB cluster PBs, EPCs, a PrePB cluster including PrePBs only being located between GC and PB clusters, and a mature PC cluster comprising BMPCs. The figure is the 3D plotting of the samples along the first 3 principal components accounting for 36.1%, 25.7% and 14% of the variance respectively. The 5 clusters comprising the populations are shown by colored clouds.

Using these 9303 unique genes, a principal component analysis (PCA), performed with GenomicScape PCA tool (S1 File), classifies the 8 populations into 8 distinct groups. Fig. 3 shows the 3D visualization of this PCA, in which each sample is plotted in the space formed by the first 3 principal components. Actually, the 8 populations can be grouped into 5 major clusters: a B-cell cluster including NBCs and MBCs, a GC cluster comprising CBs and CCs, a PB cluster formed by PBs and EPCs, a PrePB cluster including only PrePBs located between the GC and PB clusters, and a mature PC cluster grouping BMPCs. In order to identify genes driving the transition between 2 clusters of cell populations, a SAM analysis (unpaired Wilcoxon statistics, fold change ≥ 2, 300 permutations, FDR = 0%) was performed after merging the cell populations belonging to a same PCA cluster. The merging of the populations was done with GenomicScape (S1 File) and the top100 genes driving the transition between two clusters are listed in S3 Table. A majority of the top100 genes differentially expressed between BC and GC clusters encodes for proteins involved in cell cycle including *CCNB1, CDK1, AURKA, BIRC5, CDC20, ZWINT, TYMS, CCNB2, KIF2C* and *BUB1*. Genes coding for cell communication signals including *EMP1, TNFRSF1B, CD226, IGF1, IL2RA, PDGFRA, VEGFA* and *TNFRSF8* drive the transition from the GC to the PrePB clusters. The transition from the PrePB to the PB clusters is positively correlated with genes coding for cell communication signals (*ITGA6*, *CD38, SDC1, PECAM1, TNFSF8, CAV1* and *COL24A1)* and negatively with genes coding cell cycle and DNA replication (*POLE2, ORC1, CDC45, MCM10, MCM4* and *CDCA7*) (S3 Table). Genes coding for transcription factors and cell communication signals (*KLF4, CXCL12, ITGA8, VCAM1, SULF2* and *IGFBP7*) drive the transition of the PB to PC clusters.

### Visualization of genes differentially expressed between the 8 B cell and plasma cell populations

**Fig 4:**
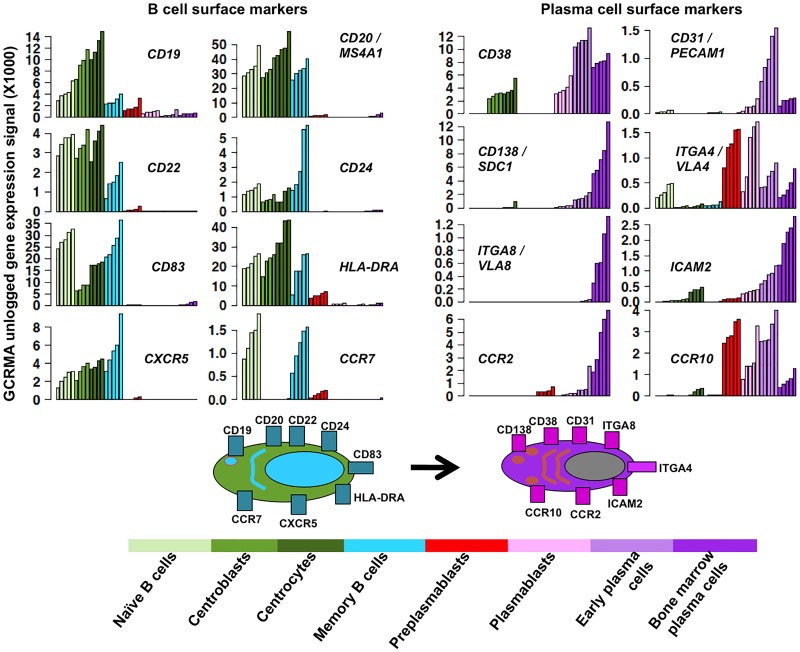
Visualization of genes coding for B cell or plasma cell surface markers. Gene expression was assayed using Affymetrix U133 plus 2.0 microarrays and data are the unlogged GCRMA-normalized expression signal of each gene in the 8 B cell to plasma cell populations. Data are the expression profiles of genes coding for B cell or plasma cell surface markers obtained with GenomicScape (see S1 File)

**Fig 5:**
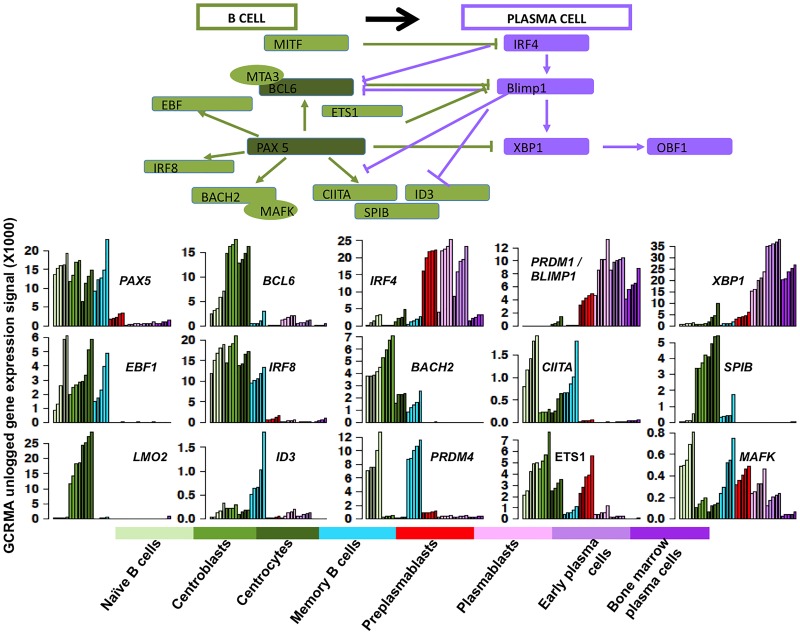
Visualization of the genes coding for B cell or plasma cell transcription factors. See the legend of Fig. 4. Data are the expression profiles of genes coding for transcription factors controlling B cell or plasma cell identities obtained with GenomicScape.

A limitation of large lists of genes is the extraction of the prominent information. GenomicScape makes it easy to visualize the expression of set of genes (up to 10000) in the 8 B and plasma cell populations and to export the figures (see S1 File). This is illustrated in Fig. 4, which provides the expression of genes coding for remarkable B cell (*CD19, CD20, CD22, CD24, CD83, HLA-DR, CXCR5, CCR7*) or plasma cell surface markers (*CD38, CD31, CD138, ITGA4, ITGA8, ICAM2, CCR2, CCR10)*, and in Fig. 5 showing the expression of genes coding for transcription factors controlling B cell or plasma cell identities (*PAX5, BCL6, EBF1, IRF8, BACH2, CIITA, SPIB, LMO2, ID3, PRDM4, ETS1, MAFK, IRF4, PRDM1/BLIMP1, XBP1*). In addition, GenomicScape makes it possible to easily determine genes coexpressed with a given gene (S1 File) and provides links with the usual bioinformatics databases, Reactome, and Gene ontology.

## Discussion

The aim of this study is to provide a free and convenient data-mining web site, which makes it possible to build a molecular atlas of the B cell differentiation (http://genomicscape.com/microarray/nbtopc.php) using publicly available transcriptome data of naïve B cells, centroblasts, centrocytes, memory B cells, preplasmablasts, plasmablasts, early plasma cells and bone marrow plasma cells without any knowledge in bioinformatics. Such an extensive description of the molecular portrait of the human B cell to plasma cell differentiation has not been reported yet. 9303 unique genes are differentially expressed between the 8 populations of human B cells to plasma cells differentiation using a SAM supervised analysis. Using principal component analysis, the 8 populations can be grouped into five major clusters: a B-cell cluster (NBCs and MBCs), a Germinal Center cluster (CBs and CCs), a Preplasmablast cluster (prePBs), a Plasmablast cluster (PBs, EPCs) and a mature plasma cell cluster (BMPCs).

Most of the genes whose expression positively drives the transition from B-cell to Germinal Center clusters are genes coding for proteins implicated in cell cycle (*CCNB1, CDK1, AURKA, BIRC5, CDC20, ZWINT, TYMS, CCNB2, KIF2C* and *BUB1*) as expected given the cell cycling status of CBs and CCs whereas NBCs and MBCs are quiescent. Expression of genes coding for proteins involved in cell communication signals (*EMP1, TNFRSF1B, CD226, IGF1, IL2RA, PDGFRA, VEGFA* and *TNFRSF8)* drives the transition of the Germinal Center to Preplasmablast clusters. The transition from PrePB to PB clusters is negatively correlated with the expression of genes coding for actors of DNA replication and cell cycle (*ORC1, RFC3, DEPDC1B, CDC45, MCM4, MCM10* and *CDCA7*), which is hardly surprising given the high proliferation rate in preplasmablasts compared to plasmablasts [5]. PrePB to PB transition is positively correlated with the expression of genes coding for cell communication signals, immunoglobulin (Ig) synthesis, transport and metabolism. Genes coding for PECAM1 (the ligand of the well-known plasma cell marker CD38) or for syndecan-1 were also found. BMPCs form a distinct cluster compared to in vitro-generated PBs and EPCs, these last two populations being tightly grouped in a PB cluster. Actually, in-vitro generated PBs and EPCs have a phenotype close to that of peripheral blood PBs and early PCs, which have to home to bone marrow or mucosa to survive and further differentiate [3, 9]. BMPCs are mature PCs as evidenced by a high expression of genes encoding for cell transporters and metabolism, indicating a need to import a lot of nutrients and produce energy in order to synthetize high levels of Ig. They also overexpressed genes coding for cell communication signals in agreement with their close requirement of bone marrow environment to survive. This “Naïve B cell to Plasma cell” Atlas allows the user to quickly visualize online whether a given protein or pathway could be involved in the process of B cell to plasma cell differentiation. It should be useful for further understanding this differentiation, and in particular the abnormalities of these processes in B and plasma cell neoplasias. Of note, recent studies have reported gene expression profiling of human plasma cells purified from the bone marrow or spleen or of in-vitro generated plasma cells using Affymetrix or Illumina microarray platforms [10, 11]. These publicly-available microarray data are available from GenomicScape, which can mine microarray data from various platforms. The current analysis can be easily extended to these other studies. In particular, the current genes differentially expressed between B and PC populations could efficiently classify the B and PC plasma cell populations reported in the study by Cocco et al. [10] using Illumina microarrays (see S1 File). In summary, GenomicScape is a flexible and free platform to easily extract biological information from genome-wide gene expression data. Additional tools for microarray data mining can be implemented, using the same aim: speed and easiness. Besides the current atlas of the transcriptome of the B cell to plasma differentiation, GenomicScape should be useful to stimulate the use of publicly available microarray data and lead to further understanding of normal and abnormal biological processes.

## Materials and Methods

### Overview of GenomicScape

GenomicScape (http://www.genomicscape.com) allows users to easily visualize and analyze publicly available gene expression data, and to explore the landscape of gene annotation resources for genes of interest. The goal of this ongoing effort is to make available public microarray data and an easy-to-use web tool to quickly analyze them. To date, 50 publicly available gene/ncRNA expression datasets from over 5000 microarray experiments performed on different cell types and obtained with Affymetrix, Illumina or Agilent platforms have been uploaded into GenomicScape, which allows the user to readily extract information from these data. Standard tools to mine microarray data have been implemented on GenomicScape and a user guide is provided in S1 File. Briefly, the “Expression/Coexpression Report” tool allows the user to search for a gene using name or other identifiers, to visualize its expression profile in cell populations/tissues and to find genes whose expression in various samples are correlated to that of a given gene. The SAM tool uses the Significance Analysis of Microarrays (SAM) algorithms to identify genes differentially expressed between groups of samples (http://statweb.stanford.edu/~tibs/SAM/). To get a quick overview of the biological value of these genes, their expression and functional annotation can be visualized and exported by clicking their gene identifier. A heat map of the expression values of top-ranked genes across different sample groups can be generated and exported. The Principal Component Analysis (PCA) tool makes it possible to find how samples can group together using metric correlations, without making any assumption (unsupervised clustering). The PCA tool provides 2D and 3D visualizations of the samples plotted along the first 2 or 3 principal components. Sample colors are assigned by default or can be chosen by the user and tables and figures can be exported.

### GenomicScape software architecture

GenomicScape uses a MySQL database (version 5.1) for storing microarray data and gene annotations from usual databases (predominantly NCBI and Ensembl). The application core functionality, was developed with PHP version 5.3 utilizing R (http://www.r-project.org/) and Bioconductor (http://www.bioconductor.org/) scripts for data-mining. The web interface is implemented with PHPBOOST web framework and uses jQWidgets javascript library.

### Cell populations and gene expression data

Peripheral blood cells from healthy volunteers were purchased from the French Blood Center (Etablissement Français du Sang Pyrénnées Méditérannée, Toulouse, France). After removal of CD2^+^ cells using anti-CD2 magnetic beads (Invitrogen, Cergy Pontoise, France), CD19^+^CD27^+^ MBCs and CD19^+^CD27^-^ NBCs were sorted with a multicolor fluorescence FACSAria device with a purity ≥ 95%. CBs, CCs and BMPCs were purified from tonsils or bone marrow of human individuals and phenotypically characterized as indicated [4, 12]. PrePBs, PBs, and EPCs were generated using a 3-step in vitro model starting from peripheral blood MBCs as reported [3, 5]. Whole genome gene expression profiling (GEP) of purified NBCs, CBs, CCs, MBCs, PrePBs, PBs, EPCs and bone marrow PCs (BMPCs) were obtained using Affymetrix U133 2.0 plus array (Affymetrix, Santa Clara, CA) in the Microarray platform of IRB (http://irb.montp.inserm.fr/en/index.php?page=Plateau&IdEquipe=6) using the same methodologies for probe amplification and microarray hybridization avoiding batch effect. GEP are publicly available from GEO database (http://www.ncbi.nlm.nih.gov/geo/, CBs and CCs: GSE15271 [4]) or from ArrayExpress database (http://www.ebi.ac.uk/arrayexpress/, BMPCs: E-MEXP-2360 [12], NBCs, MBCs, PrePBs, PBs and EPCs: E-MTAB-1771, E-MEXP-2360 and E-MEXP-3034 [3, 5].

### Data analysis

The publicly available data of these 8 B cell to plasma cell populations (38 samples) were normalized using GCRMA algorithm. They were grouped in a “Human B cells to plasma cells GCRMA” file under GenomicScape accession number GS-DT-2 available at http://www.genomicscape.com/microarray/browsedata.php?acc=GS-DT-2. Genes whose expression is differentially expressed between the 8 B cell to plasma cell populations were identified using GenomicScape SAM tool (http://genomicscape.com/microarray/webSam.php). The list of differentially expressed genes was used to perform a PCA analysis (http://genomicscape.com/microarray/pca.php). The molecular pathways, encoded by the genes differentially expressed between the naïve B cell to mature plasma cell subpopulations, were pointed out using reactome FI (Functional Interaction) cytoscape plugin (http://wiki.reactome.org/index.php/Reactome_FI_Cytoscape_Plugin). Pathways significantly enriched in a given cell subpopulations with a FDR < 0.001 are selected.

## Supporting Information

S1 File User manual of GenomicScape. (DOCX)

S1 Fig Top 20 genes differentially expressed between NBCs and MBCs, CBs and CCs, prePBs and PBs, PBs and EPCs and EPCs and BMPCs. Data are the result of SAM analysis (two class unpaired, Wilcoxon test, FDR = 0, fold change ≥, permutation = 300).
(PDF)

S1 Table Genes differentially expressed between naïve B cells (NBCs), centroblasts (CBs), centrocytes (CCs), memory B cells (MBCs), preplasmablasts (PrePBs), plasmablasts (PBs), early plasma cells (EPCs), and bone marrow plasma cell (BMPCs). Data are the SAM multiclass expression contrasts of each gene in a given cell subpopulation compared to the others.
(XLS)

S2 Table Reactome molecular pathways overexpressed by each of the 8 B cell to plasma cell subpopulations. The molecular pathways encoded by the 9303 differentially expressed genes were analyzed with Reactome functional interaction cytoscape plugin. Pathways significantly enriched in a given cell subpopulations with a FDR < 0.001 are selected.
(XLS)

S3 Table Genes driving the transition between 2 clusters of cell populations. In order to identify genes driving the transition between 2 clusters of cell populations, a SAM analysis (unpaired Wilcoxon statistics, fold change ≥ 2, 300 permutations, FDR = 0%) was performed after merging the cell populations belonging to a same PCA cluster. The top100 genes driving the transition between two clusters are listed.
(XLS)
