# Supplementary material for: GenomicScape: An Easy-to-Use Web Tool for Gene Expression Data Analysis. Application to Investigate the Molecular Events in the Differentiation of B Cells into Plasma Cells
Source: PLoS Comput Biol. 2015 Jan 29;11(1):e1004077. doi: 10.1371/journal.pcbi.1004077 (PMC4310610; doi:10.1371/journal.pcbi.1004077)
Supplement: S1 File — (DOCX) [file pcbi.1004077.s001.docx]

**Supplementary Information S1.**

**User manual**

**1. Select a dataset and sample groups to analyze.**

To select a dataset, click “**Browse Data”** in GenomicScape home page or use the following link: http://www.genomicscape.com/microarray/browsedata.php. The dataset “Human B cells to plasma cells GCRMA” used in the current study is selected by default. It also available under GenomicScape accession number GS-DT-2 (http://www.genomicscape.com/microarray/browsedata.php?acc=GS-DT-2). More than 50 publicy-available datasets comprising over 5000 microarray experiments have been presently uploaded into Genomicscape and can be used for other analyses. In the “Human B cells to plasma cells GCRMA” dataset, samples and groups have been organized according to the published biological experiments and the colours to visualize the gene expression in the groups preselected. But the user may reorganize the samples, the groups, or the colours. Each group name is preceded by a checkbox, which can be checked or unchecked to delete or add a given group in the analysis. By default, sample names are hidden. To choose the samples in a group, click “**Show sample names**” to display sample names and unselect or select samples. The user may also merge groups to define new groups. Once the groups and samples of interest have been selected, click “**Analyze the data**” at the bottom of the window. GenomicScape will display the tools to visualize or analyse the data. The tools Expression/coexpression Report, Significance of Analysis microarray, Principal Component Analysis will be used below.

**2. Run a SAM analysis using GenomicScape to identify genes differentially expressed between populations.**

To load GenomicScape SAM tool, click “**ANALYSIS TOOLS**” and then “**webSAM**” from the home page or use the link: <http://www.genomicscape.com/microarray/webSam.php>.

2.1 STEP I - Choose a dataset and the sample groups to compare:

- The dataset “Human B cells to plasma cells GCRMA” used in the current study is selected by default and is available under GenomicScape accession number GS-DT-2 (http://www.genomicscape.com/microarray/webSam.php?acc=GS-DT-2).
- Samples and groups of the “Human B cells to plasma cells GCRMA”dataset are selected by default. The user can select the samples par group and the groups to compare as well as the colors assigned to each group for gene expression visualization (see above: Select a dataset and sample groups to analyse).

**2.2 STEP II - Filter and statistical test settings**

**Three filters are available. The first one makes it possible to keep only protein coding genes. The second one allows keeping only one probe set per gene/ncRNA (with the highest SD). Indeed, some probe sets interrogating a same gene, in particular those with a low signal, may provide unrelevant information, and are generally deleted. The third one allows selecting the number of probe sets with the highest SD to study. Indeed, a majority of probe sets has a low signal or is weakly differentially expressed between populations, which weakens the identification of interesting probe sets when applying multiple testing correction.**

- **Select filtering options of interest. The default filters are chosen for the current analysis.**
- **Click “Apply” to move to the statistical test settings menu. To analyse the gene expression data of the manuscript the parameters** **Wilcoxon test**, **FDR ≤ 0%**, **Fold change ≥ 2**, **Permutation : 300** and data are **Unpaired** were used.
- Click “**Submit**”. GenomicScape computes SAM algorithm and displays the genes differentially expressed between sample groups. The result is a list of genes ranked according to the SAM score, but can be ranked according to other SAM parameters. Clicking a gene name displays the expression of this gene in the various populations and its functional annotation.
- The name of sample groups used in the analysis is displayed at the bottom of the table. Clicking a group name displays the genes overexpressed in this group compared to the others.
- Below the gene table, several clickable buttons allows to **Export the gene set**, **Analyse the gene set**, **Add Res. to MyGspace**, perform an **Heatmap of Top genes**, or **Run SAM again**.

**3. Comparison of gene expression of two populations.**

- In the <http://genomicscape.com/microarray/nbtopc.php> window, click “Analyze gene set”.

- The window indicating the various analysis tools is displayed and click “Significance of Analysis Microarrays”.

- The list of the probe sets is automatically pasted in the SAM filter and setting menu.

- STEP I. Select the dataset (here “Human B cells to plasma cells GCRMA”). Choose the two populations to compare by clicking “unselect All” and then clicking the 2 populations to compare (for example Naïve B cells and Memory B cells). Then select the probe set **filtering options. The default filters are chosen for the current analysis** and click “**Apply**”. The “Statistical test settings” window is displayed.
- STEP II. Statistical test settings. Indicate the statistical test used (Student or Wilcoxon), the FDR of the multiple testing analysis, the fold change in the expression between two populations and whether data are paired or unpaired. For the current analysis, Wilcoxon test, FDR ≤ 0%, fold change ≥ 2, permutation = 300, unpaired. Click ‘**Submit**’ to run the analysis.
- STEP III. webSAM output. GenomicScape displays the genes differentially expressed between the 2 population groups. The result is a list of genes ranked according to the SAM score, but can be ranked according to other SAM parameters.
- Clicking a gene name displays the expression of this gene in the various populations and its functional annotation.
- Below the gene table, several clickable buttons allows to **Export the gene set**, **Analyse the gene set**, **Add Res. to MyGspace**, or **Run again**.

**4. Principal Component Analysis tool.**

- To load GenomicScape PCA tool, click “**ANALYSIS TOOLS**” and then “**PRINCIPAL COMPONENT ANALYSIS**” from the home page or use the following link: <http://www.genomicscape.com/microarray/webSam.php>.
- STEP I. Select the dataset and sample groups of interest as described above in the select dataset section. To reproduce the PCA analysis of the current manuscript, click “**Analyse the gene set**” button. The list of the probe sets provided by the SAM analysis is automatically pasted in the PCA filter and setting menu. Then click “**Submit**” below the web page.
- STEP II. PCA filter and settings. If PCA is run first (this is not the case for the analysis of the current manuscript), unrelevant probe sets must be deleted. By default, the 2000 probe sets with the highest SD are kept for PCA, but this number can be modified. This filtering option is hidden when the user analyses preselected genes as described in STEP I. The user may also choose to get a movie of the 3D visualization of PCA. This option may reduce the speed of the computing.
- Click ‘**Submit**’ to run the analysis.
- PCA output. GenomicScape provides the variance provided by each principal component and 2D and 3D visualizations of the samples plotted along the first 2 or 3 principal components. Colours are assigned to each sample according to its group belonging. The figures can be exported as PDF files. GenomicScape generates also a .txt file containing PCA results. This .txt file contains data including the coordinates of the samples and genes along principal component axes, which can be used to customize PCA plots.

**5. Visualize the expression profile of a set of genes.**

GenomicScape “Gene expression/Coexpression Report” tool allows the user to search for a gene by name or other identifiers, to visualize its expression profile in the populations of the selected dataset and to find the genes correlated to a chosen gene. To use this tool, click “**ANALYSIS TOOLS**” and then “**EXPRESSION-COEXPRESSION REPORT**” from GenomicScape home page or use the following link: [http://www.genomicscape.com/microarray/expression.php](http://www.genomicscape.com/microarray/webSam.php).

The dataset “Human B cells to plasma cells GCRMA” used for the current study is selected by default.

- Select he groups and samples of interest as described above in the paragraph “Select a dataset and sample groups to analyse”.

- Enter a list of genes or probe sets (one probe set per line and maximum number = 10000)

Clicking **“Partial Match”** or **“Exact Match”** indicates to look for genes whose name either contains the typed name or exactly matches with the typed name.

- Click “**Submit**”.

- GenomicScape returns a clickable list of probe sets interrogating the gene of interest in the selected dataset. When several probe sets are available for a given gene, they are sorted from the highest to the lowest standard deviation (SD). A toolbar is available at the top of the search result to either “**Plots all the genes**” (i.e., visualize the expression of the probe set(s), “**Export the gene set**”, “**Analyse the gene set**” or “**Add list to MyGspace**”.

- To get the probe sets coexpressed with a given probe set, click it. GenomicScape displays the gene expression plot of the clicked probe set in the selected populations as a barplot. Click “**Coexpression Analysis**” below the plot, choose the correlation parameters and then click “**Submit**”. GenomicScape provides the list of genes correlated to the selected gene The heatmap and the coexpression network of the top correlated genes across selected populations are shown. From the coexpression network, the user may click a given coexpressed gene and GenomicScape will display the functional annotation and the coexpression plot of the gene.

**6. Analyzing gene data set performed with different microarray platforms.**

Once a list of genes whose expression using a microarray platform is able to discriminate various cell populations, this gene list can be used to investigate gene expression profiling of populations, performed with another microarray platform. As an example, we investigated whether the 9303 genes differentially expressed in the current 8 B cell to Plasma cell populations and obtained with an Affymetrix platform could classify gene expression of human B cells and plasma cells performed with an Illumina platform.

- In the <http://genomicscape.com/microarray/nbtopc.php> window, click “Analyze gene set”.

- The window indicating the various analysis tools is displayed and click “Principal Component Analysis”.

- Select the dataset “Cocco, In vitro generation of long-lived human Plasma Cells” and click “**Submit**” button to run the analysis.
- PCA output. GenomicScape provides the variance provided by each principal component and 2D and 3D visualizations of the samples plotted along the first 2 or 3 principal components. Colours are assigned to each sample according to its group belonging. The figures can be exported as PDF files. GenomicScape generates also a .txt file containing PCA results. This .txt file contains data including the coordinates of the samples and genes along principal component axes, which can be used to customize PCA plots.
