# Supplementary material for: GenomicScape: An Easy-to-Use Web Tool for Gene Expression Data Analysis. Application to Investigate the Molecular Events in the Differentiation of B Cells into Plasma Cells
Source: PLoS Comput Biol. 2015 Jan 29;11(1):e1004077. doi: 10.1371/journal.pcbi.1004077 (PMC4310610; doi:10.1371/journal.pcbi.1004077)

Figure S1A – Top 20 genes differentially expressed between naïve B cells and memory B cells (two class unpaired SAM analysis, Wilcoxon test, FDR = 0, fold change ≥ 2, permutation = 300)

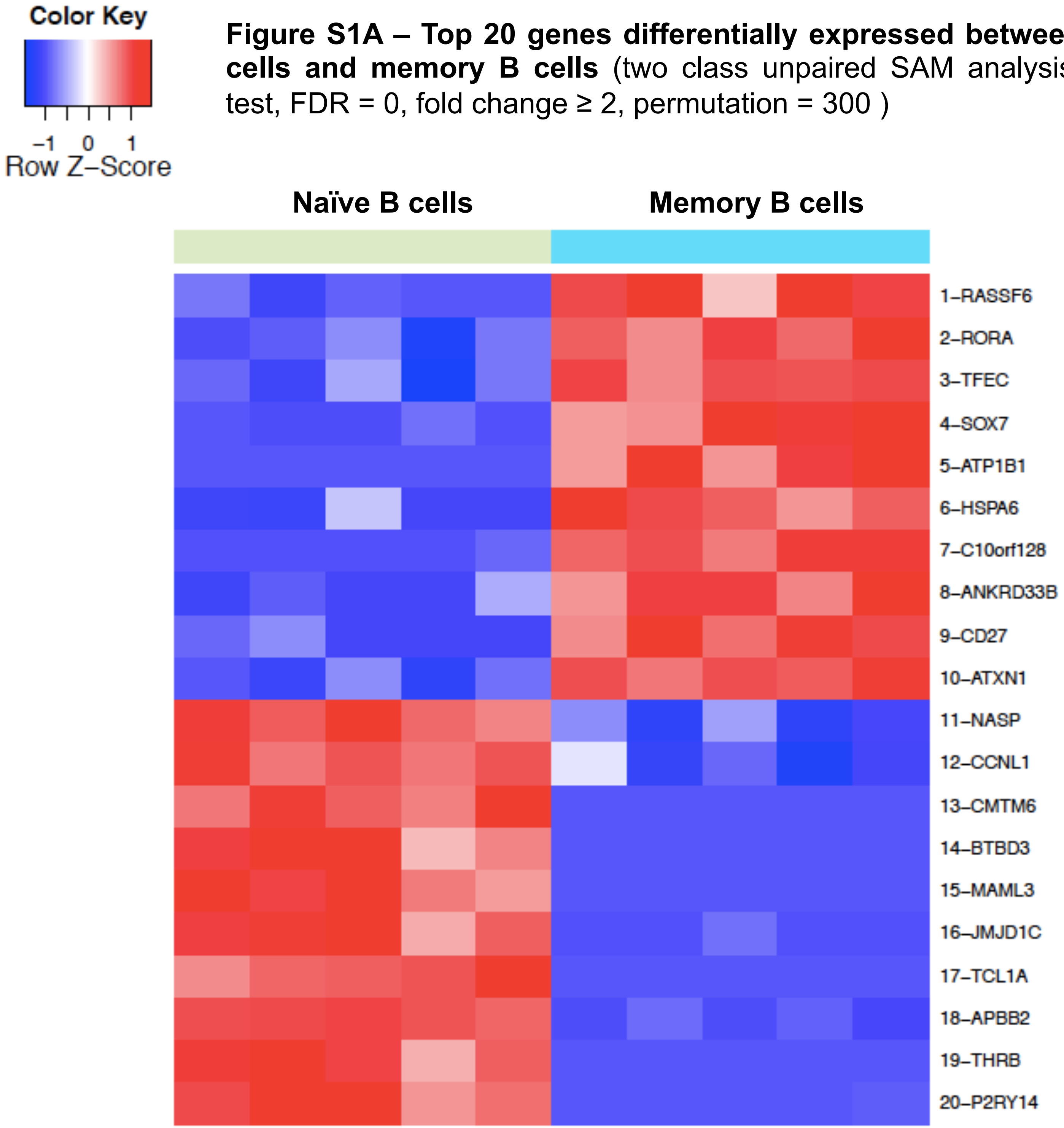

Figure S1B – Top 20 genes differentially expressed between centroblasts and centrocytes (two class unpaired SAM analysis, Wilcoxon test, FDR = 0, fold change ≥ 2, permutation = 300)

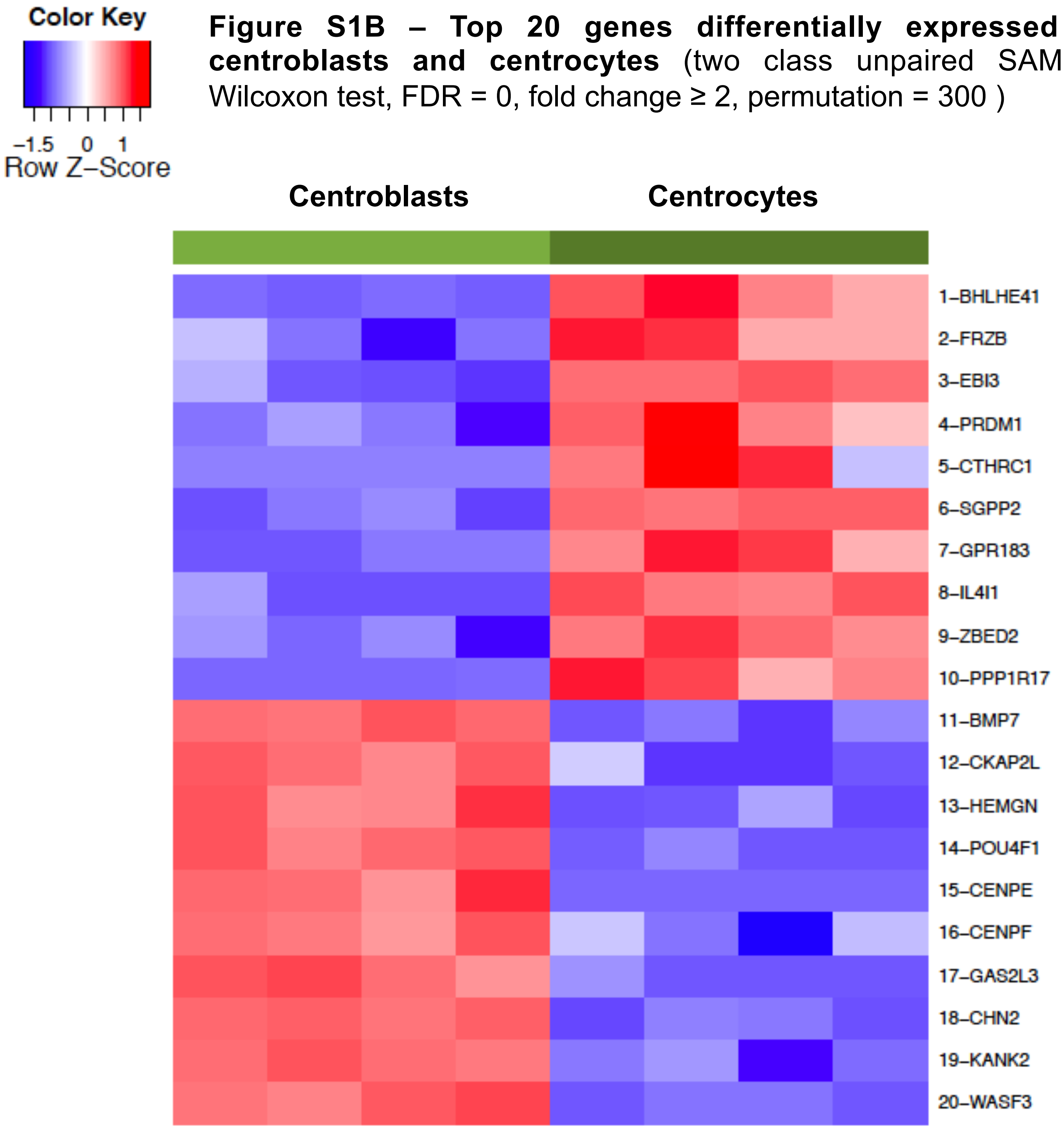

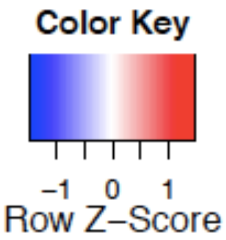

Figure S1C – Top 20 genes differentially expressed between preplasmablasts and plasmablasts (two class unpaired SAM analysis, Wilcoxon test, FDR = 0, fold change ≥ 2, permutation = 300)

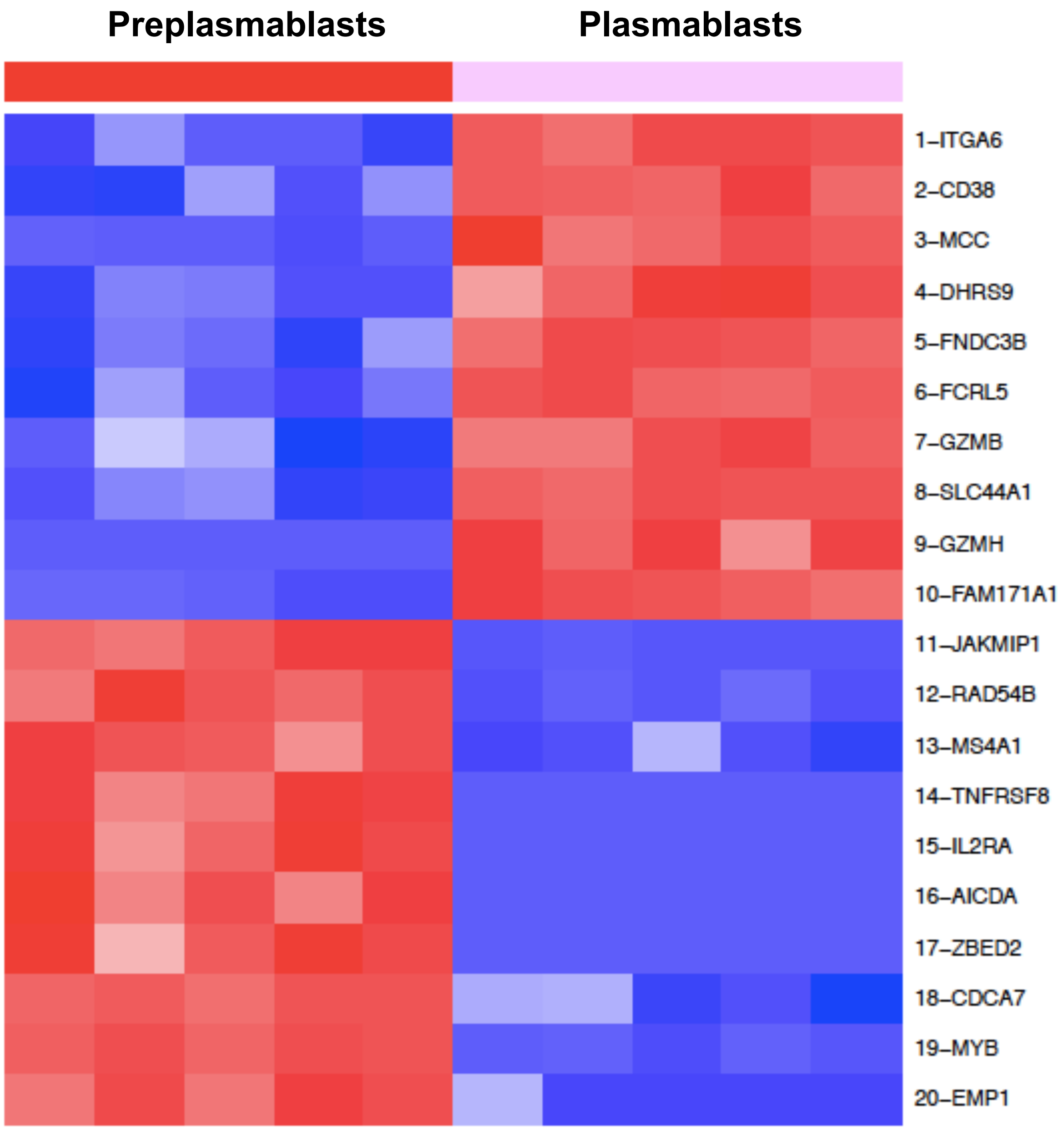

Figure S1D – Top 20 genes differentially expressed between plasmablasts and early plasma cells (two class unpaired SAM analysis, Wilcoxon test, FDR = 0, fold change ≥ 2, permutation = 300)

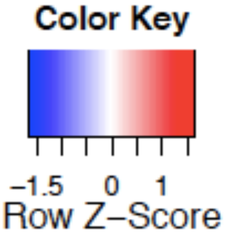

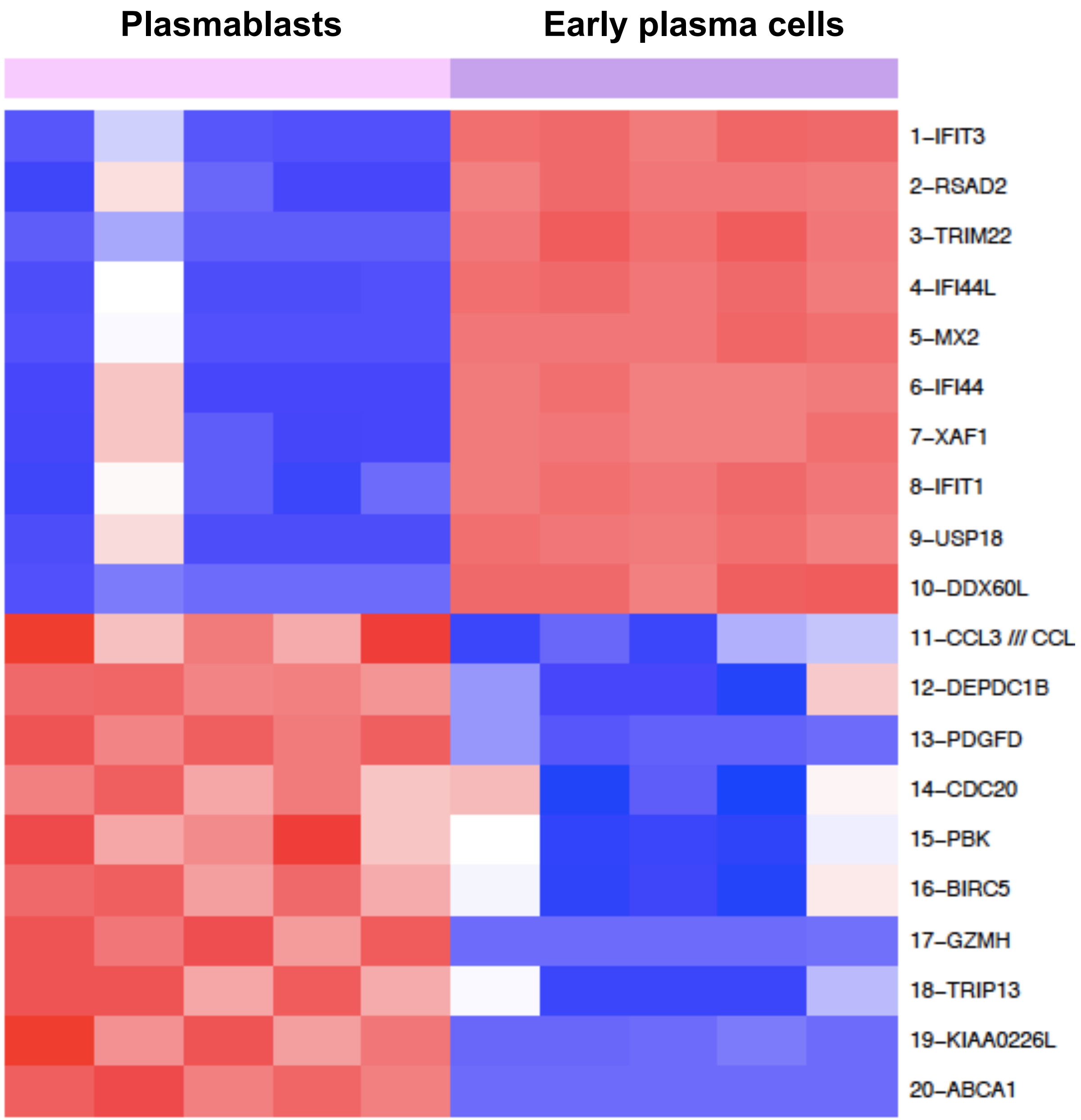

Figure S1A – Top 20 genes differentially expressed between early plasma cells and bone marrow plasma cells (two class unpaired SAM analysis, Wilcoxon test, FDR = 0, fold change ≥ 2, permutation = 300)

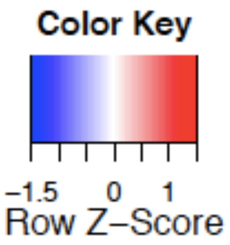

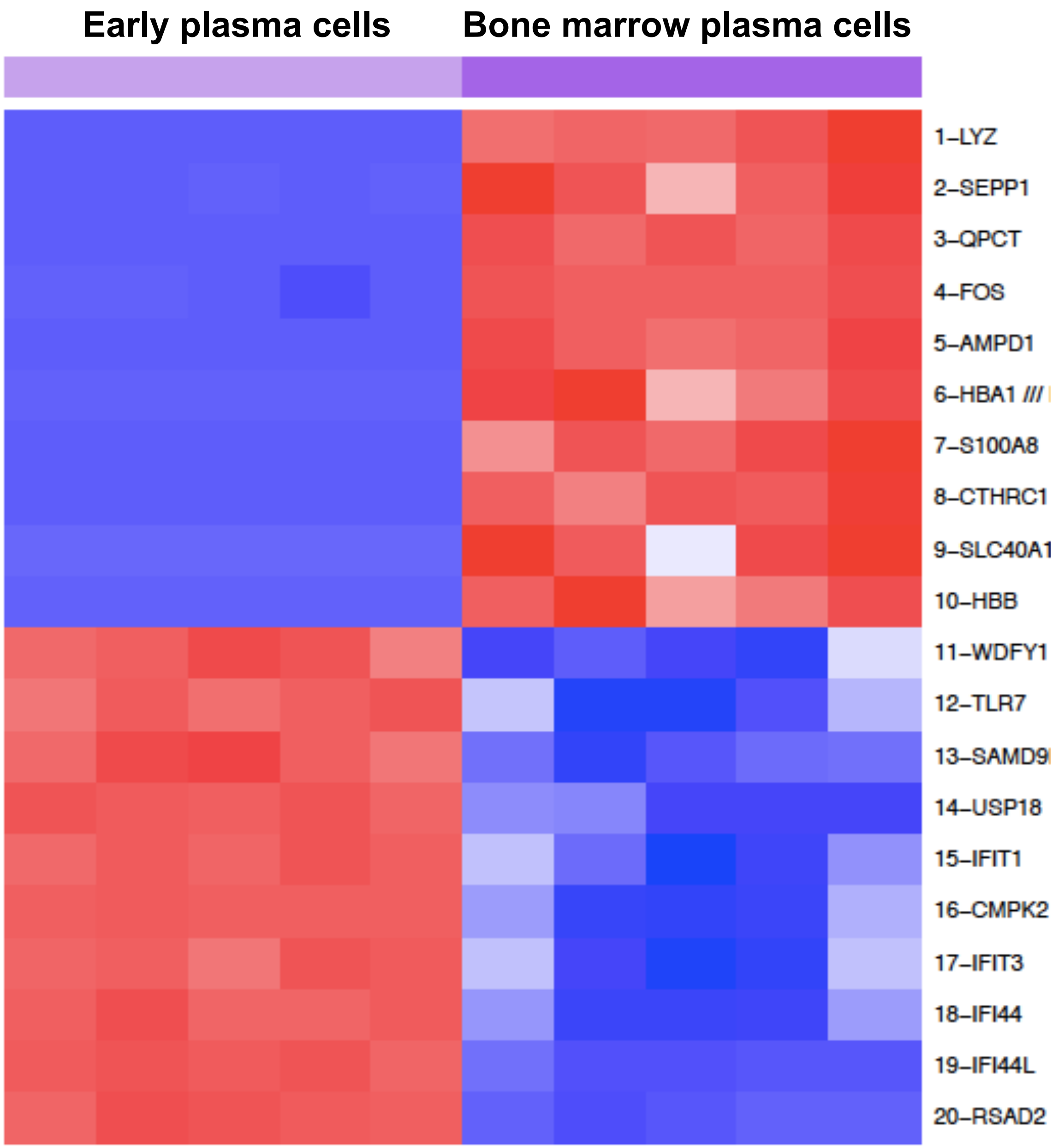

Supplement: S1 Fig — Data are the result of SAM analysis (two class unpaired, Wilcoxon test, FDR = 0, fold change ≥, permutation = 300). (PDF) [file pcbi.1004077.s002.pdf]
